# Identification of *Anaplasma ovis* appendage-associated protein (AAAP) for development of an indirect ELISA and its application

**DOI:** 10.1186/s13071-017-2297-z

**Published:** 2017-07-28

**Authors:** Zhenguo Wang, Jifei Yang, Qingli Niu, Kelly A. Brayton, Jianxun Luo, Guangyuan Liu, Hong Yin, Zhijie Liu

**Affiliations:** 1 State Key Laboratory of Veterinary Etiological Biology, Key Laboratory of Veterinary Parasitology of Gansu Province, Lanzhou Veterinary Research Institute, Chinese Academy of Agricultural Sciences, Lanzhou, People’s Republic of China; 2 Department of Veterinary Microbiology and Pathology, Washington State University, Pullman, WA 99164 USA; 3 Jiangsu Co-innovation Center for Prevention and Control of Important Animal Infectious Diseases, Yangzhou, China

**Keywords:** *Anaplasma ovis*, AAAP, ELISA, Seroprevalence

## Abstract

**Background:**

Ovine anaplasmosis is a tick-borne disease that is caused by *Anaplasma ovis* in sheep and goats. The pathogen is widely distributed in tropical and subtropical regions of the world. At present, diagnosis of the disease mainly depends on microscopy or nucleic acid based molecular tests, although a few serological tests have been applied for the detection of *A. ovis* infection.

**Results:**

Here we describe the identification of an *A. ovis* protein that is homologous to the *A. marginale* appendage-associated protein (AAAP). We expressed a recombinant fragment of this protein for the development of an indirect enzyme-linked immunosorbent assay (ELISA) for the detection of *A. ovis*. *Anaplasma ovis*-positive serum showed specific reactivity to recombinantly expressed AAAP (rAAAP), which was further confirmed by the rAAAP ELISA, which also demonstrated no cross-reactivity with sera from animals infected with *A. bovis* or other related pathogens in sheep and goats. Testing antibody kinetics of five experimentally infected sheep for 1 year demonstrated that the rAAAP ELISA is suitable for the detection of early and persistent infection of *A. ovis* infections. Investigation of 3138 field-collected serum samples from 54 regions in 23 provinces in China demonstrated that the seroprevalence varied from 9.4% to 65.3%, which is in agreement with previous reports of *A. ovis* infection.

**Conclusions:**

An *A. ovis* derived antigenic protein, AAAP, was identified and the antigenicity of the recombinant AAAP was confirmed. Using rAAAP an indirect ELISA assay was established, and the assay has been proven to be an alternative serological diagnostic tool for investigating the prevalence of ovine anaplasmosis of sheep and goats.

## Background

Ovine anaplasmosis is a tick-borne disease of sheep, goats and small ruminants caused by *Anaplasma ovis* [1–3]. *Anaplasma ovis* is a non-motile, obligate intraerythrocytic Gram-negative bacterium that belongs to the order Rickettsiales [4]. Following the reorganisation of the order in 2001, this pathogen is classified along with *A. marginale*, *A. centrale*, *A. bovis* and *A. caudatum* which infect ruminants, *A. phagocytophilum* a zoonotic agent, and *A. platys* that infects dogs [4]. Biological vectors of *A. ovis* are ticks of the genera *Dermacentor* and *Rhipicephalus* and most likely other tick species [5–9]. The study of *A. ovis* was often neglected since it is considered to be moderately pathogenic and induce only mild clinical signs [7, 10]. However, *A. ovis* infection resulting in severe disease has been reported in bighorn sheep, goats and sheep [9, 11, 12]. Although the pathogen is known to be widespread in tropical and subtropical countries, the extent of infection and the loss of livestock productivity remain poorly understood [7, 12].

The detection of *A. ovis* in livestock has traditionally been based on the identification of acute infections, using a microscopic examination of Giemsa-stained blood smears. Light microscopy is the most inexpensive and quickest laboratory test, but also the least sensitive, and is highly dependent on examiner experience [12, 13]. Moreover, it is crucial that the smears should be prepared during the early acute phase of signs and before initiation of effective antimicrobial treatments. Nucleic acids based tests, such as polymerase chain reaction (PCR), quantitative real-time PCR (qPCR), and loop-mediated isothermal amplification (LAMP) have been alternative tests for the direct detection of *A. ovis* infection in both experimental and field studies [14–16]. These methods are restricted by the limited sensitivity of the detection in persistently infected carrier animals with very low-level bacteremia [13, 17]. In contrast, serological tests have the advantage of detecting antibodies from infected animals during all stages of *Anaplasma* infection [18].

A recombinant *A. marginale* major surface protein 5 (Msp5) based competitive inhibition enzyme-linked immunosorbent assay (CI-ELISA) has been developed and shown to detect *A. marginale*-infected cattle, including persistently infected carriers [19]. This assay was later confirmed to be suitable for the detection of antibodies to *A. ovis* infected goats due to the conservation of Msp5 epitopes among *Anaplasma* strains [12, 20], and it was also found to detect antibodies from *A. phagocytophilum* and *Ehrlichia* species [21, 22]. Because of the potential for cross-reaction when using the CI-ELISA, the results need to be interpreted cautiously. In this paper, we describe the identification of an *A. ovis* antigenic protein, AAAP, and the development of an indirect ELISA for the specific detection of *A. ovis* in sheep and goats.

## Methods

### Bacteria and experimental animals

The *A. ovis* isolate used in this study was obtained from an infected sheep in Haibei County in Qinghai Province, and the blood containing live pathogens and 8% dimethyl sulfoxide (DMSO) has been cryopreserved in liquid nitrogen since 2008 at the Lanzhou Veterinary Research Institute, Chinese Academy of Agricultural Sciences.

Three-month-old sheep were purchased from a commercial farm in Jingtai County, Gansu Province. The sheep were screened for the absence of *A. ovis*, *Babesia* and *Theileria* by weekly examination of blood smear by light microscopy and previously described PCR protocols specific for each pathogen for a month before conducting animal experiments [3, 23, 24].

Sheep No. 101 was splenectomized to ensure rapid initiation and propagation of the infection by intravenously inoculating 10 ml of infected *A. ovis* cryopreserved blood (approximately 10% bacteremia). Eight sheep (Nos. 420, 470, 489, 103, 106, 134, 174 and 183) were used to collect serum.

### Preparation of serum samples

Sheep (Nos. 103, 106, 134, 174, 183) were infected by inoculating each animal with 5 ml of bacteremic blood that had been collected from sheep No. 101 when the bacteremia was approximately 10%. The serum samples were collected every 2 days for the first 15 days, followed by twice a week till 43 days, once a week till 85 days, once 2 weeks till 181 days and once a month till a year period. Sheep (Nos. 420, 470, 489) were infected by inoculation of infected blood from sheep No. 101 twice in two-week intervals for hyperimmune serum preparation. The serum samples were prepared immediately after *A. ovis* was observed in the thin blood smears. Positive sheep sera against *A. bovis*, *Mycoplasma ovipneumoniae*, *Mycoplasma capricolum capricolum*, *Babesia motasi*, *Babesia* sp. Xinjiang, *Theileria uilenbergi* and *Theileria luwenshuni*, and positive yak sera against *A. marginale* were obtained from previous collections and stored at -20 °C in our laboratory.

Serum samples from sheep (Nos. 103, 106, 134, 174, 183, 420, 470, 489) before infection were used as negative controls. An additional 434 negative samples were obtained from experimental animals purchased from 2009 to 2016. These animals were determined to be free of *A. ovis*, *Babesia* and *Theileria* spp. as described above.

**Fig. 1:**
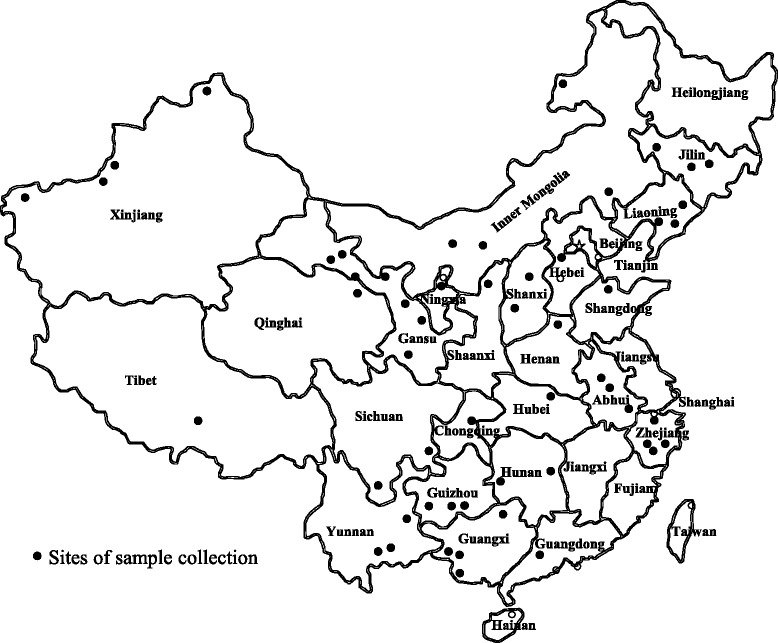
Detection of *A. ovis* infection in sheep and goats from 23 provinces in China

Field samples (*n* = 3138) were randomly collected from domestic sheep and goats from 54 different locations in 23 provinces between 2010 and 2016 (Fig. 1). All samples were collected in non-anticoagulation tubes, and the serum was separated and stored at -20 °C in our laboratory.

### DNA specimens

Whole blood was taken from the jugular vein of each experimentally infected animal and collected in a sterile tube containing an anticoagulant (ethylene diamine tetraacetie acid, EDTA). DNA was extracted from the blood using a genomic DNA extraction kit (Qiagen, Hilden, Germany) according to the manufacturer’s instructions.

### Bacterial purification

The venous blood from sheep No. 101 (10% bacteremia) was harvested in a sterile flask containing anticoagulant (EDTA). The red blood cells were separated by centrifugation at 1000× *g* for 10 min, and the upper layer containing the white blood cells was discarded. The packed red blood cells were suspended in phosphate-buffered saline (PBS, pH 7.2), and then the remaining white blood cells were removed using a commercial leucocyte filter (Nanjing Shuangwei Biotechnology, Nanjing, China). The flow-through was centrifuged as above, and the supernatant was discarded. The harvested red blood cells were suspended in four volumes of PBS containing 7% glycerin and placed at room temperature for 30 min, and then centrifuged again to harvest the red blood cells. The cells were then added to a flask containing four volumes of physiological saline to let the cells lyse completely. The lysate was centrifuged at 1000× *g* for 10 min to get rid of cell debris. The supernatant was then centrifuged at 10,000× *g* for 30 min to collect the bacterial pellet. The pellet was washed three times with physiological saline by centrifugation at 10,000× *g* for 10 min. The white pellet at the bottom of the tube was the purified bacteria, which were then stored at -70 °C till use.

### Immunoprecipitation and mass spectrometric analysis

Fifteen μg sepharose beads (CNBr-activated Sepharose™ 4B, GE Healthcare Life Sciences, Beijing, China) were added to 500 μl 0.1 mM HCl and gently mixed for 15 min. Agarose was pelleted by centrifugation at 12,000× *g* at room temperature for 10 s, and then re-suspended in 600 μl washing buffer (0.1 mM HCL) and divided into 6 aliquots of 100 μl in 1.5 ml tubes. Equal amounts of sheep (Nos. 420, 470, 489) sera before and after infection was added into each tube respectively and incubated at room temperature for 30 min with gentle shaking. The agarose and antibody conjugates were pelleted by centrifugation at 3000× *g* at 4 °C for 2 min, and the conjugates were washed three times using washing buffer. The purified bacteria were lysed using RIPA lysis buffer (Beyotime, Beijing, China), and 200 μl of bacterial lysates containing approximately 500 μg of antigen were added to the conjugates and incubated at 4 °C overnight with gentle shaking. The samples were centrifuged to collect immunoprecipitation complexes at 3000× *g* at 4 °C for 2 min. The complexes were washed three times using washing buffer. The antibody-antigen conjugates were eluted by washing with 20 μl elution buffer (100 mM Glycine, pH 2.5). The resulting samples were used for sodium dodecyl sulfate-polyacrylamide gel electrophoresis (SDS-PAGE) analysis. The separated bands were digested with trypsin at 37 °C overnight. Peptides were extracted with 50% acetonitrile (ACN, Fisher Chemical, Shanghai, China) containing 5% formic acid (FA, Fluka, Shanghai, China), followed by 100% ACN. The peptides were dried and then resuspended in 2% ACN containing 0.1% FA. The peptides were then identified using liquid chromatography-electrospray ionisation tandem mass spectrometry (LC-ESI-MSMS) (Triple TOF 5600, AB SCIEX, Concord). Resulting values for monoisotopic peaks were analysed using the computer program Mascot [25]. The sequences obtained from the mass spectrometry were used to identify the full-length open reading frame by search and alignment against an ongoing genome sequencing project for *A. ovis* strain Haibei (GenBank accession no. CP015994).

### Cloning of the truncated *aaap* gene

PCR primers were designed based on the *aaap* gene sequence from the *A. ovis* strain Haibei genome sequence. The restriction sites, *EcoR* I and *Hind* III were introduced into the 5′ and 3′ primers, respectively. The primers were aaap-F: 5′-CCG GAA TTC AGG GTA CTG GTA ATG GGC-3′ and aaap-R: 5′-CCC AAG CTT CTA AAT AGC AAG ACT TTG CGT ATT AG-3′. Genomic DNA from an infected blood sample from sheep No. 101 served as a template for the PCR.

The PCR had a total volume of 25 μl containing 12.5 μl Premix *Taq*™ (TaKaRa *Taq*™ Version 2.0 plus dye), 0.5 μl of each primer (20 μM), 2.0 μl of template DNA, and 9.5 μl of distilled water. The cycling conditions were as follows: 4 min of denaturation at 94 °C, 35 cycles at 94 °C for 1 min, annealing at 55 °C for 30 s, and 72 °C for 1 min, with a final extension step at 72 °C for 10 min. The PCR products were cloned into the pGEM-T-Easy Vector (Promega, Beijing, China), according to the manufacturer’s instructions, and then digested using *EcoR* I and *Hind* III restriction enzymes (New England Biolabs, Hitchin, UK). The resulting fragment was subsequently cloned into the pET-30a expression vector (Novagen, Shanghai, China) using the same restriction sites. The correct insertion of the *aaap* gene fragment was confirmed by sequencing (Sangon Biotech Company, Shanghai, China).

### Expression and purification of the recombinant AAAP protein

The recombinant plasmid pET-30a-P35 was transformed into BL21 *E. coli* (DE3 strain). The cells were cultured in LB medium at 37 °C for 6 h and expression was induced by addition of 1 mM isopropyl-β-d-thiogalactoside (IPTG) when the optical density (OD) reached 0.6. The bacterial cultures were harvested and lysed by ultrasonication in binding buffer (20 mM imidazole, 20 mM sodium phosphate, 0.5 M NaCl, 8 M Urea, pH 7.6) and then purified as inclusion bodies from *E. coli* cells. The target protein was purified with the AKTA design system (Amersham Bioscience, Uppsala, Sweden) using 5 ml HiTrap FF crude. The column was washed with 3–5 column volumes of distilled water and then equilibrated with at least 5 column volumes of binding buffer. The flow rate was 2 ml/min for 5 ml columns. The pretreated sample was applied using a syringe pump, and then the column was washed with binding buffer (80 mM imidazole, 20 mM sodium phosphate, 0.5 M NaCl, 8 M Urea, pH 7.6) until the absorbance reached a steady baseline. The sample was eluted with elution buffer (250 mM imidazole, 20 mM sodium phosphate, 0.5 M NaCl, 8 M Urea, pH 7.6) until the absorbance reached a steady baseline.

### Preparation of AAAP specific rabbit immune serum

Two New Zealand white rabbits were immunised three times by injecting with 200 μg of recombinant AAAP protein at 2-week intervals. For the first immunisation, the recombinant AAAP protein was emulsified with Freund’s complete adjuvant (FCA) (Sigma-Aldrich, Shanghai, China) at a ratio of 1:1. For the remaining immunisations, AAAP was emulsified with incomplete Freund’s adjuvant at a ratio of 1:1. Serum samples were collected 2 weeks after the last immunisation, and stored at -20 °C until use.

### Western blotting analysis

Optimal amounts of the recombinant protein AAAP and the crude antigen (Bacterial lysate) were separated in SDS-PAGE using 12% polyacrylamide gels under reducing conditions and transferred to nitrocellulose (NC) membranes. The NC membranes were blocked with 5% skimmed milk powder in 0.1 M Tris-buffered saline (pH 7.6) containing 0.1% Tween-20 (TBST) at 4 °C overnight. To verify the expression and purification of the recombinant protein AAAP, the RGS-His™ mouse anti-histidine antibody (1:4000, Qiagen, Hilden, Germany) and secondary alkaline phosphatase (AP) conjugated goat anti-mouse IgG + IgM (H + L) antibody (1:10,000, Dianova, Hamburg, Germany) were used to detect the His-tag on the recombinant protein. To test the antigenicity and specificity of recombinant proteins, 1:100 diluted sheep serum samples positive for *A. ovis*, *A. bovis*, *M. ovipneumoniae*, *M. capricolum capricolum*, *B. motasi*, *Babesia* sp. Xinjiang, *T. uilenbergi*, *T. luwenshuni*, as well as negative control serum from uninfected sheep were used as primary antibody and 1:5000 diluted AP conjugated monoclonal anti-goat/sheep secondary antibody (Sigma-Aldrich) were used. To detect native AAAP, pre-immunization rabbit serum and rabbit AAAP antiserum were tested with the crude antigen on western blot. The secondary antibody was AP-conjugated goat anti-rabbit immunoglobulin antibody (1:5000, Sigma-Aldrich). All of the serum samples and the secondary antibodies were diluted in dilution buffer (TBST containing 1% bovine serum albumin, pH 7.2). Binding of secondary antibody was detected with 5-bromo-4-chloro-3-indolyl phosphate (BCIP)/ nitroblue tetrazolium (NBT) substrate (Sigma-Aldrich). The approximate molecular weights of the presented protein bands were calculated by comparing their migrations with the standard Protein Ladder (Thermo Scientific, Beijing, China).

### ELISA

A checkerboard titration was used to determine the concentration of coating antigen (1.5, 2.0, 2.5, 3.0 μg/ml), serum (1:50, 1:100, 1:200, 1:400 dilutions) and conjugate (1:15,000, 1:20,000, 1:25,000 dilutions). The optimum conditions were set as follow. Briefly, the plates (JET BIOFIL, Canada) were coated with 2.5 μg/ml of recombinant protein AAAP in 0.1 M carbonate/bicarbonate buffer, pH 9.6, at 4 °C overnight. After 3 washes with PBS containing 0.1% Tween 20 (PBST), the plates were blocked with 100 μl of 1% gelatin in carbonate/bicarbonate buffer at 37 °C for 1 h. After 3 washes with PBST, the plates were incubated at 37 °C for 1 h with 100 μl of the positive and negative sera (1:100) which were distributed in duplicate. The positive sera were a mixture from hyperimmune sheep 420, 470, 489 2 weeks after second infected-blood inoculation as described above and the negative serum was from sheep 420 before infected-blood inoculation. The plates were washed with the same procedure and incubated with 100 μl of secondary antibody (anti-goat/sheep IgG-peroxidase, Sigma-Aldrich, 1:20,000) in PBST at 37 °C for 1 h. The plates were washed 3 times, and 100 μl of 3,3′,5,5,’-tetramethylbenzidine (TMB, KPL, 52–00-03) was added to each well and incubated at 37 °C for 10 min. The reaction was stopped by adding 100 μl of 2 M H_2_SO_4_ and the OD (450 nm) values were read using an ELISA automat (Bio-Rad, California, USA). The specific antibody mean rate (AbR%) was calculated for each serum sample with the following formula, AbR% = (Sample mean OD – Negative control mean OD)/(Positive control mean OD – Negative control mean OD) × 100%.

## Results

### Identification of *A. ovis aaap*

**Fig. 2:**
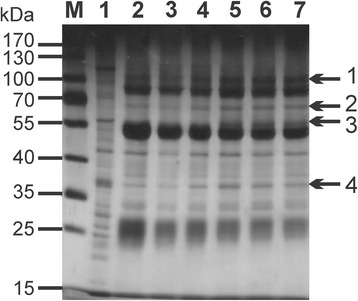
Silver staining of SDS-PAGE gel for analysis of *A. ovis* lysates immune- precipitation. Lane 1: *A. ovis* lysates; Lanes 2–4: *A. ovis* lysates immunoprecipitation with sera from pre-immunized sheep (Nos. 420, 470, 489); Lanes 5–7: *A. ovis* lysates immunoprecipitation with sera from *A. ovis* infected sheep (Nos. 420, 470, 489). Numbers at right side indicate four differently expressed bands, and band 4 was predicted to be AAAP

**Fig. 3:**
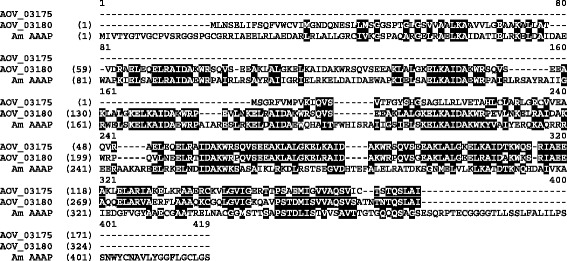
Alignment of AAAP sequences. The deduced amino acid sequences of the full length AAAP gene (AOV_3180) and the partial gene (AOV_3175) from *A. ovis* are shown aligned with the original AAAP protein sequence from *A. marginale* (Am AAAP)

We performed immunoprecipitation assays using *A. ovis* bacterial protein extracts and serum samples collected from *A. ovis* infected animals. As a control, serum from animals before infection was used with the same bacterial extracts. In the immunoprecipitation assay, four bands were detected as a novel or at higher densities in the group immunoprecipitated with the positive sera as compared with the control group immunoprecipitated with the negative sera as shown in Fig. 2. These bands were further analysed using mass spectrometry, and the resulting peptide sequences were BLASTed against the *A. ovis* genome. Band 4 was identified as *aaap*, which corresponds to AOV_03180 with an open reading frame of 972 bp in size. The translated protein contains 323 amino acids with a predicted molecular weight of 35.5 kDa. The *aaap* sequence has been deposited in GenBank with accession number KY670611. There is a second gene in the genome in tandem with AOV_03180 that has similar features, designated AOV_03175, which appears to be a truncated version of *aaap* (Fig. 3). The deduced amino acid sequence of *aaap* showed 31% identity to the appendage-associated protein of *A. marginale* (AAAP; AM878; GenBank accession no. AAV86790) (Fig. 3).

**Fig. 4:**
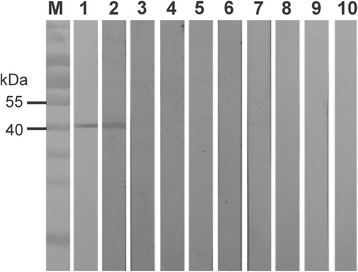
Specificity and assessment of cross-reactivity of recombinant AAAP using Western blot*.* Lane M: molecular weight marker; Lanes 1–11 had 5 μg of purified rAAAP blotted, which was then reacted with the primary antibodies as follows: Lane 1: mouse anti-RGS-His antibody; Lane 2: *A. ovis*-positive sheep serum; Lane 3: serum from uninfected sheep; Lane 4–9, 11: positive sheep sera against *Mycoplasma ovipneumoniae*, *M. capricolum capricolum*, *Babesia motasi*, *Babesia* sp. Xinjiang, *Theileria uilenbergi*, *T. luwenshuni* and *A. bovis*, respectively; Lane 10: positive cattle serum against *A. marginale*

A truncated *aaap* fragment encoding 299 amino acids (aa 25–323) was cloned into the pET-30a expression vector for recombinant protein expression. The pET-30a-P35 plasmid was expected to express an rAAAP protein with a molecular weight of 40.0 kDa. When the rAAAP was tested for reactivity with *A. ovis*-positive serum samples, a clear band of the appropriate size was observed, while no cross-reactivity was seen with serum samples containing antibodies to *M. ovipneumoniae*, *M. capricolum capricolum*, *B. motasi*, *Babesia* sp. Xinjiang, *T. uilenbergi*, *T. luwenshuni*, *A. bovis* or negative serum samples from healthy sheep (Fig. 4). This result indicated that the *aaap* gene encodes a potential antigenic protein of *A. ovis*.

**Fig. 5:**
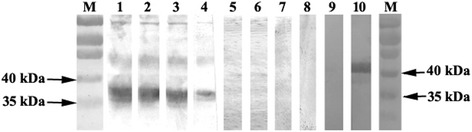
Identification of native AAAP form the different weight lysate of *A. ovis* in Western blot. Lane M: molecular weight marker; Lanes 1–8: blotted with *A. ovis* lysate (Lanes 1 and 5: 20 μg; Lanes 2 and 6, 15 μg; Lanes 3 and 7, 10 μg; Lanes 4 and 8, 5 μg); Lanes 9–10: blotted with recombinant AAAP, reacted with rabbit anti-rP35 antibody; Lanes 1–4, 10, reacted with rabbit anti-rAAAP antibody; Lanes 5–9, reacted with pre-immunized rabbit serum

To identify native AAAP protein in *A. ovis*, rabbit anti-rAAAP protein serum was prepared and used in Western blot analysis with purified *A. ovis* lysates. Both native AAAP protein in the lysates and the rAAAP protein were recognised by the rabbit anti-rAAAP sera, while no reaction was observed when preimmune rabbit sera were used (Fig. 5). The molecular weight of native AAAP appeared lower than rAAAP in the Western blot, most likely due to an extended protein structure of rAAAP leading to a retarded migration during electrophoresis (34, 35). These data confirmed the *A. ovis* origin and antigenicity of the AAAP protein.

### Establishment of the rAAAP indirect ELISA

The rAAAP based indirect ELISA was eventually established with 100 μl of 2.5 μg/ml rAAAP protein, 100 μl of a 1:100 dilution of each serum sample to be tested, and 100 μl of 1:20,000 diluted secondary antibody in each well in the reaction system. These conditions were used in all subsequent experiments.

**Fig. 6:**
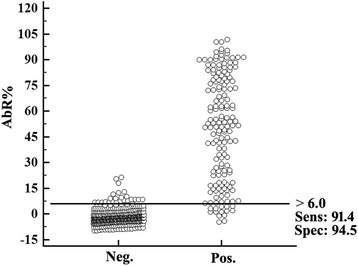
Determination of ELISA cut-off value. The cut-off value of the rAAAP ELISA was determined to be 6.0% by testing 434 negative and 163 positive reference sera using interactive dot diagram. With this threshold, the calculated sensitivity and specificity were 95.1 and 95.4%, respectively

The cut-off value of the ELISA was determined using interactive dot diagram (MedCalc software, version 11.4.2.0) [26] by testing 597 reference sera (434 negative sera and 163 positive sera). It was defined using the percentage of the specific antibody mean rate (AbR%), which was eventually set to be 6.0%. With this threshold, 25 false positive and 14 false negative sera were detected, resulting in a calculated sensitivity and specificity of 91.4% (95% confidence interval, CI: 86.0–95.2) and 94.5% (95% CI: 91.9–96.4), respectively (Fig. 6).

**Fig. 7:**
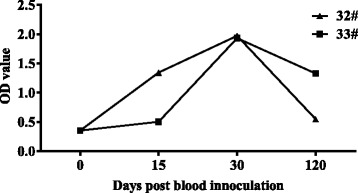
Evaluation of the cross-reactivity of the rAAAP ELISA. Cross-reactivity with *A. marginale-*positive sera collected from yaks (Nos. 32, 33) by time course after infected blood inoculation

**Fig. 8:**
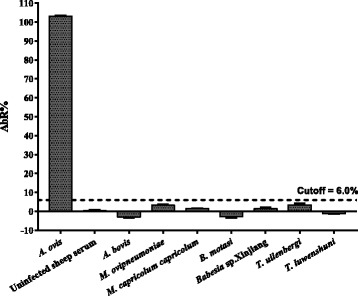
Evaluation of the specificity of the rAAAP ELISA. Reaction with positive sheep sera against *A. ovis*, *A. bovis*, *Mycoplasma ovipneumoniae*, *M. capricolum capricolum*, *Babesia motasi*, *Babesia* sp. Xinjiang, *Theileria uilenbergi*, *T. luwenshuni* and negative serum from uninfected sheep, respectively

The specificity of the rAAAP indirect ELISA was evaluated using control samples, which were used previously in Western blot analysis and yak serum samples of *A. marginale*. The positive results were detected with the *A. ovis*-positive, and *A. marginale*-positive serum (Fig. 7), and no cross-reactivity was seen with the serum samples from *A. bovis*, *M. ovipneumoniae*, *M. capricolum capricolum*, *Babesia* sp. Xinjiang, *B. motasi*, *T. uilenbergi*, or *T. luwenshuni* (Fig. 8).

### The kinetics of antibody response in experimentally infected sheep

**Fig. 9:**
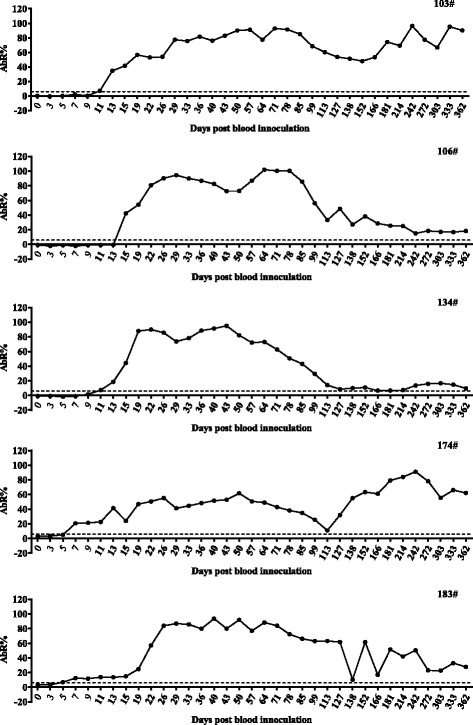
Antibody kinetics for AAAP. Five sheep (Nos. 103, 106, 134, 174 and 183) infected with *A. ovis* were monitored for a year period, and the antibody response was tested by rAAAP ELISA

The serum samples from five experimentally infected sheep (Nos. 103, 106, 134, 174, and 183) were collected at different time points during infection. These samples were used to test the kinetics of antibody response against rAAAP using the established ELISA (Fig. 9). A significant increase of antibodies against rAAAP was observed after the sheep were infected. However, the earliest antibody response differed from 5 to 13 days post-infection between individual animals. From then on, a sharp increase of antibody response was observed, and the infected animals typically retained high antibody titers for approximately 100 days, when antibody a cycling pattern of decreasing and increasing antibody titers appeared in some of the animals (especially Nos. 103, 174). Moreover, the antibody response could be detected a year after infection, indicating that a test is a suitable tool for monitoring persistent *A. ovis* infections.

### Detection of the field samples with the rAAAP indirect ELISA

The rAAAP indirect ELISA tested the field samples. The results showed that the mean positive rate was 35.3% (1106/3138) with the highest positive rate of 66.7% (66/99) in Yunnan Province and the lowest rate of 9.4% (8/85) in Henan Province (Table 1).

**Table 1:** Seroprevalence of *A. ovis* infection tested using rAAAP indirect ELISA in field samples from 23 provinces

Province	Region	No. of sera	No. of positive sera	Positive rate (%)
Anhui	Chaohu	34	18	52.94
	Hefei	143	64	44.76
	Guangde	15	5	33.33
Chongqing	Wanzhou	23	3	13.04
Gansu	Lintan	98	31	31.6
	Dingxi	28	4	14.9
	Yongchang	74	13	17.57
	Jiayuguan	81	2	2.47
	Jiuquan	18	1	5.56
	Jingtai	69	9	13.04
	Sunan	46	6	13.04
Guangdong	Zhaoqing	37	19	51.35
Guangxi	Guilin	79	34	43.04
	Jingxi	35	11	31.43
	Tianyang	25	8	32.00
	Pingxiang	18	3	16.67
Guizhou	Guiyang	95	6	6.32
	Qinglong	29	22	75.86
	Rongjiang	34	23	67.65
Hebei	Baoding	151	17	11.26
Henan	Anyang	85	8	9.41
Hubei	Suizhou	68	9	13.24
Hunan	Xiangtan	26	14	53.85
	Xinhuang	29	8	27.59
Inner Mongolia	Chifeng	134	42	31.34
	Manzhouli	13	3	23.08
	Baotou	11	0	0
	Bayan Nur	11	4	36.36
Jilin	Changchun	27	7	25.93
	Qianan	14	2	14.29
	Yongji	26	13	50.00
Liaoning	Anshan	27	17	62.96
	Dandong	28	5	17.86
	Huanren	29	7	24.14
Ningxia	Wuzhong	81	27	33.33
Qinghai	Qilianshan	98	64	65.31
Shaanxi	yulin	74	17	22.97
Shandong	Dongying	90	38	42.22
Shanxi	Lvliang	50	19	38.00
	Xinzhou	195	98	50.26
Sichuan	Hejiang	31	16	51.61
	Panzhihua	31	13	41.94
Tibet	Lhasa	113	46	40.71
Xinjiang	Akesu	92	16	17.39
	Yili	287	153	53.31
	Habahe	100	60	60.00
	Kashi	20	1	5.00
Yunnan	Honghe	30	14	46.67
	Fuyuan	35	27	77.14
	Yanshan	34	25	73.53
Zhejiang	Hangzhou	52	33	63.46
	Jinghua	15	0	0
	Lishui	15	0	0
	Taizhou	35	1	2.86
Total		3138	1106	35.3

## Discussion

Ovine anaplasmosis has been neglected perhaps due to knowledge gaps on its pathogenicity, morbidity, mortality, clinical signs and economic losses. The causative agent, *A. ovis*, is considered as a moderate pathogen typically inducing only subclinical signs [4, 7]. However, an exception was found in sheep and goats in Ejinaqi, Western Inner Mongolia in China, where the morbidity of the disease was as high as 40–50% and the mortality was 17%, and the clinical signs such as anemia, jaundice and emaciation were observed [9]. *Anaplasma ovis* infection causing severe disease has also been reported in bighorn sheep, domestic sheep and goats in North America and Africa [11, 12]. The *A. ovis* Haibei strain used in this study caused several deaths in sheep herds in Haibei County in Qinghai Province in 2008. In addition, when healthy and splenectomized sheep were inoculated with infected blood from the Haibei strain the animals died. With the development and application of DNA-based tests such as conventional PCRs, specific qPCRs [16, 27], more and more studies have demonstrated high infection rates of *A. ovis* in North America, Europe, Africa, the Middle East and Asia, which have been well summarized by Renneker et al. [7]. These data indicate that more attention should be paid to the economic impact and health implications of *A. ovis* infection in small ruminants.

Two types of serological tests have been applied for analysis of *A. ovis* infection including complement fixation (CF) and a recombinant *A. marginale* Msp5 based competitive inhibition ELISA (CI-ELISA) [9, 10, 12, 28]. Although the *A. marginale*-derived CI-ELISA will successfully detect antibodies of *A. ovis* infection in sheep and goats, the fact that the Msp5 B-cell epitope is conserved among *Anaplasma* species [21, 29] means that the results need to be interpreted cautiously due to the potential for co-infections with other *Anaplasma* species in sheep and goats [30–33]. Further, the CI-ELISA cannot be used to quantitatively evaluate antibody titers, in the manner of a direct ELISA. A lack of knowledge of *A. ovis*-specific antigens has restricted the development of a species-specific test. Although a few *A. ovis* antigens, including Msp2, Msp3 and Msp4 have been reported [3, 34, 35], none of them have been developed into an *A. ovis*-specific serological test. In the present study, we identified the *aaap* gene from the *A. ovis* genome from mass spectrometry data. Recombinant AAAP showed a specific reaction with *A. ovis*-positive sera in both Western blot and ELISA analysis, while no cross-reactivity was observed with positive serum of *A. bovis* and other related agents. However, cross-reactivity with *A. marginale*-positive sera occurred in the rAAAP ELISA, most likely due to the presence of similar AAAP amino acid sequences in both *A. ovis* and *A. marginale*, such as multiple imperfect peptide repeats centred around the sequence ELKAIDA [36]. Rabbit anti-rAAAP serum was able to detect native AAAP on Western blots of purified *A. ovis* lysates from infected blood, which revealed multiple protein bands with a molecular size around 35 kDa. That is consistent with the fact that *A. ovis* contains tandemly duplicated copies of *aaap* in its genome [37]. Although a few studies have reported the infection of *A. marginale* in wild ruminant species such as bighorn sheep, white-tailed deer, etc. [3, 38], *A. marginale* infection of sheep and goats was not found in China and most of the world, which indicates that the AAAP indirect ELISA has the potential to be applied for establishing species-specific diagnostic assays for sheep and goats. However, only limited *A. bovis* serum samples and none of the serum samples of *A. phagocytophilum*, *A. capra* as well as *Ehrlichia* spp. were included in the present study, further evaluation of the specificity of the rAAAP ELISA method are needed.

In this study, an indirect ELISA was established using rAAAP for detection of antibodies to *A. ovis* infection. The accuracy of an ELISA test is dependent on the cut-off value used to classify samples as seropositive or not, and changing the cut-off value can change the results of the test [38, 39]. The cut-off for the rAAAP-ELISA was determined to be 6.0% (AbR%) when the minimal total number of diagnostic errors (false positives plus false negatives) was calculated after testing 434 negative and 163 positive reference sera. This is the most direct approach in defining the optimal cut-off for a serological test [38, 39]. With this threshold, the sensitivity and specificity of the rAAAP ELISA were calculated to be 91.4 and 94.5%, respectively.

The prospective use of the rAAAP ELISA in detecting *A. ovis* infection was verified by testing the antibody kinetics for 1 year in five experimentally infected sheep. The test could detect an antibody response 5 to 15 days post-infection, with this time frame being in agreement with the biological features of *Anaplasma* infection, which usually takes one to several weeks to establish infection [9, 40]. After the infection was established, a sharply increasing antibody response appeared, and the high antibody titer lasted for around 3 months. A persistent antibody titer was detectable until the end of the experiment using the rAAAP ELISA. These results demonstrated the potential usefulness and applicability of this ELISA for detecting early infection and monitoring persistent *A. ovis* infection. In addition, a fluctuating antibody titer was seen during persistence in sheep Nos. 103, 106, 134 and 174, but was not so apparent in sheep No. 183. This is in line with two patterns of persistent bacteremia in *A. ovis* infected goats. The first pattern was characterised by cyclic fluctuation, similar to the pattern described for *A. marginale* infected cattle, while the bacteremia levels were relatively constant in the second pattern [34, 41, 42]. Whether these phenomena are related to the antigenic variation of the *msp2* and *msp3* multigene families resulting in cyclic rickettsemia during *A. ovis* persistent infection remains unknown [34], although this pattern has been demonstrated in *A. marginale* [41, 42].

With the established rAAAP ELISA, a large-scale study of 3138 sera from sheep and goats collected from 54 different locations in 23 provinces was undertaken. As a result, the seroprevalence of *A. ovis* infection was detected in almost all of the sampled regions, except Baotou in Inner Mongolia, and Jinghua and Lishi in Zhejiang Province. Negative results for these three regions are most likely due to the limited sample size because the presence of *A. ovis* infection has been demonstrated in Inner Mongolia and Zhejiang in our previous studies [9, 31]. A wide distribution of *A. ovis* infection in the investigated regions likely reflects the true situation in China, since the existence of *A. ovis* infection in most of these provinces has been reported recent years [30, 31, 43, 44].

## Conclusion

An *A. ovis* derived antigenic protein, AAAP, was identified in the present study. The antigenicity of the recombinant AAAP was confirmed by testing *A. ovis*-positive sera and rabbit anti-rAAAP serum. Using rAAAP an indirect ELISA assay was established, and the assay has been proven to be an alternative serological diagnostic tool for investigating the prevalence of ovine anaplasmosis of sheep and goats. However, this method may be not specific for indicating exposure to *A. ovis*. Thus further studies are needed to characterise the *aaap* gene in other related pathogens and systematically evaluate the detection ability of the rAAAP ELISA for future application in the field.

## Abbreviations

AAAP: *Anaplasma* sp. appendage-associated protein
AbR%: The specific antibody mean rate
AP: Alkaline phosphatase
BCIP: 5-bromo-4-chloro-3-indolyl phosphate
CAN: Acetonitrile
CF: Complement fixation
CI-ELISA: Competitive inhibition enzyme-linked immunosorbent assay
DMSO: Dimethyl sulfoxide
EDTA: Ethylene diamine tetraacetie acid
ELISA: Indirect enzyme-linked immunosorbent assay
FA: Formic acid
FCA: Freund’s complete adjuvant
IPTG: Isopropyl-β-d-thiogalactoside
LC-ESI-MSMS: Liquid chromatography-electrospray ionisation tandem mass spectrometry
Msp5: Major surface protein 5
NBT: Nitroblue tetrazolium
OD: Optical density
PBS: Phosphate-buffered saline
PCR LAMP: Loop-mediated isothermal amplification
PCR: Polymerase chain reaction
qPCR: Quantitative real-time PCR
SDS-PAGE: Sodium dodecyl sulfate-polyacrylamide gel electrophoresis
TBS: Tris-buffered saline
